# Identification of a novel p53 target, COL17A1, that inhibits breast cancer cell migration and invasion

**DOI:** 10.18632/oncotarget.18433

**Published:** 2017-06-09

**Authors:** Varalee Yodsurang, Chizu Tanikawa, Takafumi Miyamoto, Paulisally Hau Yi Lo, Makoto Hirata, Koichi Matsuda

**Affiliations:** ^1^ Laboratory of Clinical Genome Sequencing, Department of Computational Biology and Medical Sciences, Graduate School of Frontier Sciences, The University of Tokyo, Minato, Tokyo, Japan; ^2^ Laboratory of Molecular Medicine, Human Genome Center, Institute of Medical Science, The University of Tokyo, Tokyo, Japan

**Keywords:** COL17A1, p53, breast cancer, invasion, metastasis

## Abstract

*p53* mutation is a marker of poor prognosis in breast cancers. To identify downstream targets of p53, we screened two transcriptome datasets, including cDNA microarrays of MCF10A breast epithelial cells with wild-type *p53* or *p53*-null background, and RNA sequence analysis of breast invasive carcinoma. Here, we unveil ten novel p53 target candidates that are up-regulated after the induction of p53 in wild-type cells. Their expressions are also high in breast invasive carcinoma tissues with wild-type *p53*. The GO analysis identified epidermis development and ectoderm development, which *COL17A1* participates, as significantly up-regulated by wild-type *p53*. The COL17A1 expressions increased in a p53-dependent manner in human breast cells and mouse mammary tissues. Reporter assay and ChIP assay identified intronic p53-binding sequences in the *COL17A1* gene. The MDA-MB-231 cells that genetically over-express *COL17A1* gene product exhibited reduced migration and invasion *in vitro*. Similarly, *COL17A1* expression was decreased in metastatic tumors compared to primary tumors and normal tissues, even from the same patients. Moreover, high *COL17A1* expression was associated with longer survival of patients with invasive breast carcinoma. In conclusion, we revealed that *COL17A1* is a novel p53 transcriptional target in breast tissues that inhibits cell migration and invasion and is associated with better prognosis.

## INTRODUCTION

Breast cancer is the most common cancer worldwide in women and contributed more than 25% of the total number of new cancer cases diagnosed in 2012 [1]. The mutation frequency of the tumor suppressor *p53* is relatively low in breast cancer compared to other solid tumors [2]; however, this mutation is the second most frequent genetic alteration observed in 30-35% of breast cancer cases [3, 4]. A mutation in *p53* is also associated with aggressive subtypes, i.e., 12% in luminal A, 32% in luminal B, 75% in HER2, and 84% in triple-negative tumors [3]. Previous studies have shown that *p53* mutation is an independent marker of poor prognosis in breast cancers [5] and is also associated with the response to specific treatment regimens in breast cancer [6]. p53 functions as a transcription factor, and its target genes are involved in several pathways, including cell stemness, extracellular matrix maintenance, cell adhesion, and cell motility [7]. Thus, the dysregulation of p53 targets *via* p53 inactivation may be related to the poor prognosis of breast cancer patients. We have previously identified a number of p53 targets and elucidated the molecular mechanism by which p53 regulates apoptosis, the cell cycle, senescence, iron metabolism, and post-translational modifications [8–10]. However, the role of p53 in breast carcinogenesis has not been fully elucidated. Thus, the identification of p53 targets in breast tissues is important to understand the pathogenesis of breast cancer.

*COL17A1* encodes Collagen XVII (COL17; formerly known as BP180 or BPAG2), a transmembrane protein. COL17 is an essential component of type I hemidesmosomes (HDs) and functions as a cell-matrix adhesion molecule [11]. COL17 is highly expressed in tissues with a prominent epithelial component, including the mammary gland [12]. Autoimmunity to COL17 and mutations in *COL17A1* result in blistering skin diseases caused by a loss of attachment between the epidermis and the underlying basement membrane [13, 14]. Type I HDs were observed in normal epithelial cells but were lost in cancer cells, including invasive breast cancer [15], and pancreatic ductal epithelium [16]. COL17 has been previously reported as a down-regulated protein signature in stage II compared to premalignant cells and in premalignant cells compared to normal myoepithelial cells [17]. Although recent studies have suggested a role of COL17 in cell migration [18–20], its function in breast carcinogenesis has never been investigated. Here, we revealed *COL17A1* as a novel downstream target gene of p53 that is suppressed in breast cancer tissues with a *p53* mutation.

## RESULTS

### Correlation of COL17A1 and *p53* status

**Figure 1:**
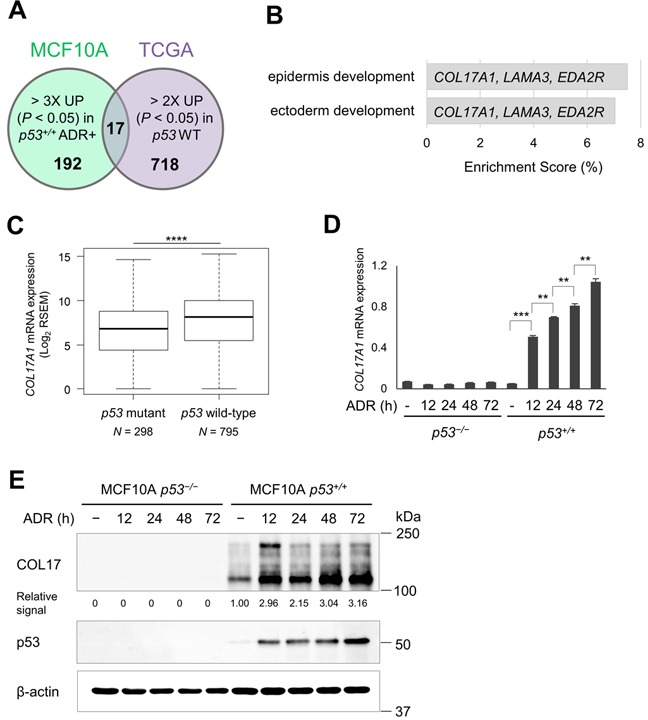
Correlation of COL17A1 and *p53* status **A.** The p53 targets were screened using MCF10A microarray analysis and invasive breast carcinoma (TCGA). A Venn diagram shows the number of genes that satisfied the inclusion criteria from each analysis and the 17 overlapping genes. Inclusion criteria: MCF10A, > 3-fold up-regulated with *P* < 0.05 by doxorubicin (ADR+) in *p53* wild-type cells (*p53*+/+); TCGA, > 2-fold up-regulated with *P* < 0.05 in *p53* wild-type (WT) tumors (see Material and methods for details). **B.** GO biological processes of the 17 overlapping gene set with *P <* 0.05 and enrichment score > 5% are shown in the bar chart with indicated participating genes. **C.** Differential mRNA expression of *COL17A1* in *p53* wild-type and mutant tumors. *N*, number of tissues. **D.** The MCF10A cells were treated with 0.5 μg/ml of ADR for 2 hours. Total RNA was isolated at 12, 24, 48 and 72 hours after ADR treatment. The qPCR result shows the relative mRNA expression of *COL17A1* normalized by *B2M* in MCF10A cells bearing *p53* wild-type (*p53*+/+) or knockout (*p53*^−/−^), with or without ADR; times (hours) indicate period after ADR treatment. **E.** Western blot analyses of COL17 and p53 in MCF10A cells treated as described in Figure 1D. β-actin is shown as a loading control. The relative signal of western blot represents protein level of combined 180-kDa and 120-kDa COL17 quantified by ImageJ and normalized by β-actin. Two-tailed Student's *t*-test; *P < 0.05, **P < 0.01, ***P < 0.001, ****P < 0.00024 (0.05/209).

To identify p53 targets in breast tissues, we performed transcriptome analysis using non-tumorigenic breast epithelial cell lines with or without wild-type *p53* (MCF10A *p53*^+/+^ and MCF10A *p53*^−/−^, respectively). MCF10A *p53*^+/+^ and MCF10A *p53*^−/−^ cells were treated with 0.5 μg/ml of doxorubicin (trade name Adriamycin^®^, ADR). Total RNA was isolated at 12, 24, and 48 hours after ADR treatment and then subjected to cDNA microarray analysis. We identified 209 genes that were up-regulated more than 3-fold (*P* < 0.05) at 12, 24, or 48 hours after ADR treatment in MCF10A *p53*^+/+^ cells compared to MCF10A *p53*^+/+^ cells without ADR treatment or MCF10A *p53*^−/−^ cells at any timepoint (Figure 1A). Second, we used the data obtained from a breast invasive carcinoma cohort (BRCA), TCGA. We selected 735 genes that were up-regulated more than two-fold in breast cancer tissues with wild-type *p53* compared to those with mutant *p53* (*P* < 0.05, Figure 1A). The 17 overlapping genes in the two analyses, including 7 reported p53 target genes, is shown in Table 1, and those results are displayed in Supplementary Figures 1 and 2. Among them, 16 genes except *SYTL2* exhibited significant association even after Bonferroni correction (*P* < 0.05/209) using the number of genes those showed significant association in the first screening (MCF10A *p53*^+/+^ and MCF10A *p53*^−/−^ cells), indicating low possibility of false positive associations. Therefore, this set of genes is likely to be regulated by p53 both *in vitro* and *in vivo*.

**Table 1:** List of 17 p53 target gene candidates from the screen (Figure 1A)

Gene	Description	MCF10A		TCGA		Accession No.	Reference PMID*
		FC^a^	*P*-value^b^	FC^c^	*P*-value^b^		
ACER2*	alkaline ceramidase 2	7	0.0320	2	1.83E-27	NM_001010887	26943039
BTG2*	BTG anti-proliferation factor 2	17	0.0382	3	3.47E-44	NM_006763	11814693, 8944033
*COL17A1*	collagen type XVII alpha 1 chain	7	0.0284	3	1.44E-04	NM_000494	
*CROT*	carnitine O-octanoyltransferase	4	1.80E-05	2	5.56E-06	NM_001243745	
EDA2R*	ectodysplasin A2 receptor	5	2.21E-05	2	1.51E-46	NM_001242310	19543321
*FAM198B*	family with sequence similarity 198 member B	78	0.0154	2	2.36E-14	NM_016613	
GDF15*	growth differentiation factor 15	52	0.0036	3	1.35E-12	NM_004864	17276395
GLS2*	glutaminase 2	51	0.0438	3	3.41E-21	NM_013267	20351271
*GREB1*	growth regulation by estrogen in breast cancer 1	13	0.0260	8	8.25E-23	NM_014668	
*LAMA3*	laminin subunit alpha 3	4	0.0405	3	4.88E-08	NM_198129	
*MICALCL*	MICAL C-terminal like	4	0.0457	2	3.88E-10	NM_032867	
*NPY1R*	neuropeptide Y receptor Y1	4	0.0346	5	2.34E-13	NM_000909	
*SLC27A2*	solute carrier family 27 member 2	4	0.0342	3	3.55E-09	NM_003645	
SPATA18*	spermatogenesis associated 18	45	0.0437	3	1.52E-37	NM_145263	21300779
*SYTL2*	synaptotagmin like 2	5	0.0337	3	0.0042	NM_032943	
TNFRSF10C*	TNF receptor superfamily member 10c (*TRID*, *TRAIL-R3*)	162	0.0080	2	6.71E-23	NM_003841	10435597
*TSPAN1*	tetraspanin 1	8	0.0203	4	5.57E-09	NM_005727	

^a^Fold-change, the up-regulated fold expression after ADR in *p53* wild-type cells (see calculation in Material and methods).

^b^Two-tailed Student's *t*-test *P-*value.

^c^Fold-change, the increased fold expression in wild-type compared to mutant *p53* tumors.

* Reported p53 downstream target gene and the reported reference study.

The overlapping genes from the two analyses were further investigated for enriched biological process using Gene Ontology (GO) enrichment analysis (Figure 1B). Epidermis development and ectoderm development were the most and second most significant GO terms, respectively. *COL17A1, LAMA3,* and *EDA2R* were included in both terms. *EDA2R* (ectodysplasin A2 receptor, *XEDAR*) is a known p53 target in several cell types including cells in the breast [21, 22]. *LAMA3* encodes a subunit of laminin-5 (Laminin-332) whose expression is altered by mutant p53 in a breast cell line [23]. A positive correlation between p53 level and the transcription level of *COL17A1* has been suggested by high-throughput analyses [24, 25], although direct regulation of *COL17A1* by p53 was not investigated previously.

Our screening indicated a strong correlation between *COL17A1* and *p53* (Figure 1C, Supplementary Figures 1 and 2). Next, we verified the induction of mRNA and protein expression of COL17A1 in MCF10A *p53*^+/+^ cells at several points of time after ADR treatment (Figure 1D and 1E). The p53 levels were gradually induced in accordance with time after ADR treatment (Figure 1E). Similarly, the COL17A1 levels increased with time after ADR treatment in a p53-dependent manner. The qPCR and western blot analyses supported the screening results that COL17A1 expression was regulated by p53.

### The p53-dependent induction of COL17A1 in human cells and mouse tissues

**Figure 2:**
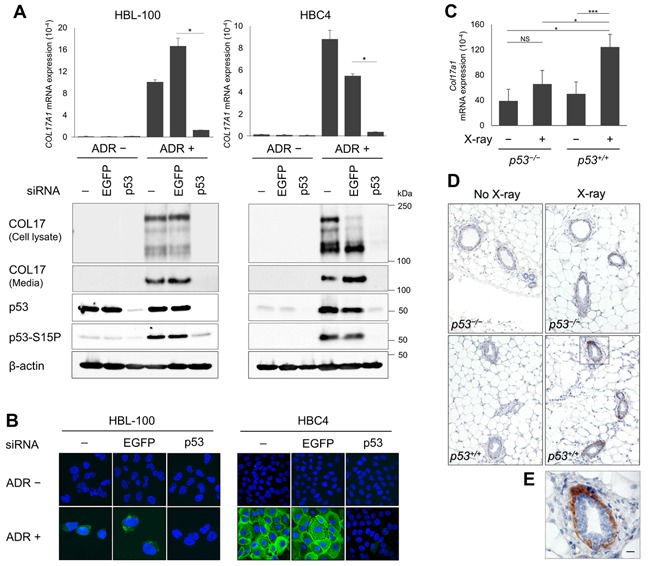
The p53-dependent induction of COL17A1 in human cells and mouse tissues **A.** The qPCR and western blot results of COL17A1 in *p53* wild-type breast cell lines, HBC4 and HBL-100, transfected with siRNA of either EGFP or p53, with or without doxorubicin (ADR) treatment. The cells were treated with ADR for 2 hours. ADR concentrations were 1 μg/mL for HBL-100 and 2 μg/mL for HBC4. Total RNA and protein were isolated at 48 hours after ADR treatment. siRNA of EGFP was used as a control. Relative mRNA expression of *COL17A1* was normalized to *ACTB*; *n* = 3. Immunoblotting results of cell lysates with antibodies against COL17 (anti-Collagen XVII), p53, and p53-S15P (p53 phosphorylated at serine 15). Cell media were blotted with anti-Collagen XVII. β-actin is shown as a loading control. **B.** Immunocytochemistry of HBL-100 (40× magnification) and HBC4 (20× magnification) cells treated as described in Figure 2A. The cells were stained with DAPI (blue) and anti-Collagen XVII (green). **C.** Relative mRNA expression of *Col17a1* normalized to *Gapdh* in mouse mammary tissues of *p53* knockout (*p53*^−/−^) or *p53* wild-type (*p53*^+/+^) mice; with or without X-ray irradiation; *n* = 3. The mice were sacrificed at 24 hours after X-ray. **D.** Immunohistochemistry results of mammary tissues staining with anti-Collagen XVII; *n* = 3 mice per group. Images were obtained at 10× magnification. **E.** The inset of Figure D, *p53*^+/+^ mice with X-ray at 40× magnification. Scale bar, 20 μm. Two-tailed Student's *t*-test; *P < 0.05, **P < 0.01, ***P < 0.001; NS, *P* ≥ 0.05.

Next, we further investigated the relationship between COL17A1 and p53 using human breast cancer cell lines and mouse breast tissues. ADR treatment in HBC4 human breast cancer cells induced an accumulation of p53 protein (Figure 2A). Although p53 protein was not increased in HBL-100 cells (Figure 2A), most likely due to a high basal expression of p53 as previously described [26], phosphorylation of p53 at Ser15, a marker of p53 activation [27], was clearly induced by ADR (Figure 2A). Next, we treated these cells with siRNA against p53 (sip53). Consistent with p53 expression, COL17A1 mRNA and protein expression levels were significantly induced by ADR, and this induction was diminished by sip53 in both cell types (Figure 2A and 2B). We detected both the 180-kDa full-length form of COL17 and its 120-kDa extracellular C-terminal domain (ectodomain) in the cell lysates, whereas only the 120-kDa form was detected in the media (Figure 2A). The ectodomain was generated by constitutive shedding of the full-length form and was released from the cell surface into media [28]. The notably decreased 180-kDa form, with a concomitant increased 120-kD ectodomain, was observed in HBC4 cells treated with ADR and siEGFP (Figure 2A), as observed in keratinocytes [29]. This increased ectodomain shedding can be activated by proinflammatory cytokines [30] which have been reported to be non-targeted induced in some siRNA-treated mammalian systems [31].

In the mouse experiment, 10-week-old *p53*^+/+^ and *p53*^−/−^ mice were exposed to 10 Gy X-ray irradiation. Twenty-four hours after irradiation, mammary tissues were collected for qPCR analysis and immunohistochemistry. The COL17A1 mRNA and protein levels were significantly induced by X-ray in *p53*^+/+^ but not *p53*^−/−^ mice (Figure 2C and 2D). In the mouse mammary gland, elevated COL17 protein levels were detected in myoepithelial cells surrounding secretory luminal cells (Figure 2E) regarding its role as a cell-matrix adhesion molecule. These data demonstrated that COL17A1 expression depends on p53 and is induced by ADR in human breast cell lines and by X-ray in mouse mammary tissues.

### *COL17A1* is a direct target of p53

**Figure 3:**
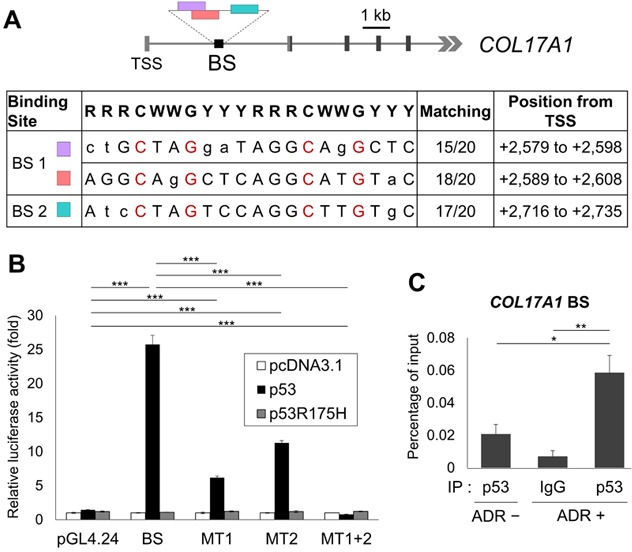
Identification of the p53 binding site **A.** Genomic structure of the human *COL17A1* gene indicates potential p53 binding sites (color boxes) in the BS construct (black box). Light gray box, untranslated region; dark gray box, translated region; TSS, transcription start site. The table shows the BS sequences aligned with the consensus p53 binding sequence (20 bases): R, purine; W, A or T; Y, pyrimidine. Identical nucleotides to the consensus are written in capital letters with the number of matching (n/20). The red-labeled nucleotides were mutated to thymine to examine the specificity of BS after mutagenesis. BS1 was mutated to MT1; BS2 was mutated to MT2; MT1+2 is double mutation of BS1 and BS2. **B.** Luciferase assay of the BS construct, the mutated BS construct (MT1, MT2, MT1+2), and the empty vector (pGL4.24) using H1299 cells co-transfected with control vector (pcDNA3.1), wild-type p53 (p53), or mutant p53 (p53R175H). Luciferase activity is indicated relative to the activity of the control vector (pcDNA3.1); *n* = 3. **C.** ChIP assay of non-treated or ADR-treated HBC4 cells of which DNA-protein complexes were then immunoprecipitated with the indicated antibodies followed by qPCR. Anti-mouse IgG was used as a negative control. The graphs show qPCR results indicating the amount of genomic fragments containing p53-binding sequences in *COL17A1* using BS primers. Two-tailed Student's *t*-test; *P < 0.05, **P < 0.01, ***P < 0.001.

To examine whether *COL17A1* is a direct target of p53, we surveyed the genomic sequence of the *COL17A1* gene and found two putative p53 binding sites, BS1 and BS2, within the first intron (Figure 3A). The DNA fragment was amplified using primers covering both BS1 and BS2 regions and then cloned into pGL4.24 vectors (BS) to evaluate the p53-dependent transcriptional activity using a reporter assay. The luciferase activity of the BS construct was markedly enhanced by co-transfection with wild-type p53 but not with mutant p53, an Arg-to-His substitution at p53 amino acid 175 (p53R175H) (Figure 3B). Next, we introduced mutations at each binding site, resulting in mutations of BS1 (MT1) and BS2 (MT2) (Figure 3A). MT1 and MT2 significantly reduced the luciferase activity compared to that of BS, although a high signal was still detected due to the existence of another non-mutated BS. The luciferase activity of MT2 when co-transfected with p53 was superior to that of MT1 (Figure 3B), suggesting that p53 binds strongly to BS1, most likely because BS1 contains long binding sequences (30 bases) and matched highly to the consensus p53 binding sequence (Figure 1A). Moreover, after two binding sites had been mutated (MT1+2), the luciferase activity was entirely drop to the control level (Figure 3B), suggesting the complete deletion of p53 binding. To further confirm whether p53 can bind to this DNA segment, we performed a chromatin immunoprecipitation (ChIP) assay using HBC4 cells with or without ADR treatment. After precipitation with an anti-p53 antibody, a DNA fragment containing p53 binding sites was quantified by qPCR. The p53 binding site in *WAF1,* a p53 target gene, was examined as a positive control region (Supplementary Figure 3). The qPCR result using *COL17A1* BS primers indicated that the endogenous wild-type p53 significantly binds stronger to *COL17A1* in ADR-treated cells compared to non-treated cells (Figure 3C). Taken together, our results suggest that p53 directly regulates *COL17A1* through p53-binding sites located in the first intron.

### The role of COL17 in tumor metastasis suppression

**Figure 4:**
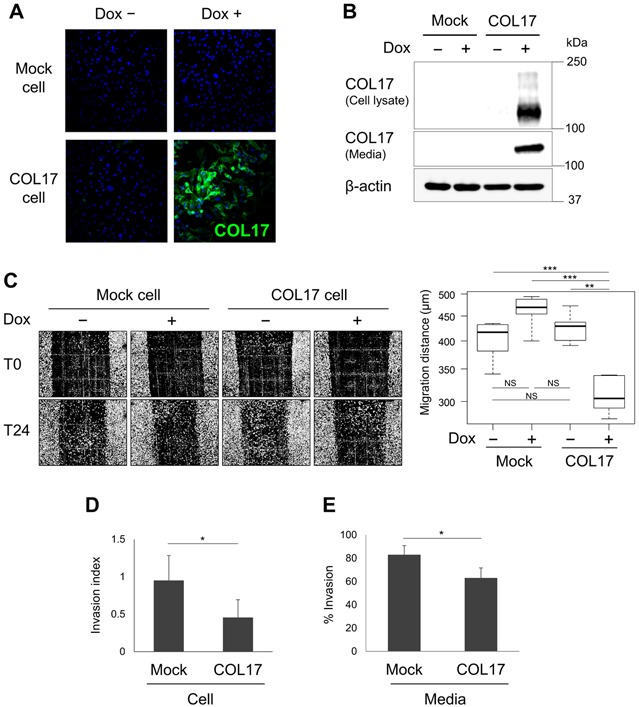
The role of COL17 in tumor metastasis suppression MDA-MB-231 cell line stably overexpressing COL17 and mock cells were used for all experiments. Cells were not treated (Dox−) or treated with 1 μg/mL of doxycycline (Dox+) for 48 hours before analysis. **A.** Immunocytochemistry of the stable cells stained with anti-Collagen XVII. **B.** Western blot of the stable cell lysates and media extracts blotting with anti-Collagen XVII. **C.** Images of the scratch region taken immediately after the scratch (T0) and 24 hours later (T24) using a phase-contrast microscope with 10× magnification; *n* = 6 per group. Migration distances from T0 to T24 were measured at the reference points (*n* = 3) using ImageJ, and the average was calculated and used for the box plot analysis. **D.** Invasion assay of stable cells analyzed at 24 hours after plating. The number of invading cells was quantified and calculated as the % invasion through the Matrigel over control chambers. The invasion index displays a proportion of the % invasion for the Dox+/Dox− condition. **E.** Media assay using parental MDA-MB-231 cells plated with conditioned media harvested from the stable COL17 or mock cell culture dishes. The parental cells invasive ability was displayed as the % invasion through the Matrigel over the control. Two-tailed Student's *t*-test; *P < 0.05, **P < 0.01, ***P < 0.001; NS, *P* ≥ 0.05.

Regarding the function of COL17 as a cell-matrix adhesion molecule [11], we examined its role in tumor metastasis using a highly invasive MDA-MB-231 cell line expressing mutant p53. We generated MDA-MB-231 cell stably expressing COL17 in the presence of doxycycline (Dox) and control cells using the tetracycline-regulated lentiviral expression system. In the absence of Dox, the Tet repressor suppresses COL17 expression (Figure 4A and 4B, Dox−). By contrast, after the addition of Dox into cell culture media for 48 hours, the 180-kDa form of COL17 is expressed, with the shed 120-kDa ectodomain form that is predominantly detected in the culture media (Figure 4A and 4B). These stable cell lines were used for subsequent experiments to investigate cell migration and invasion *in vitro*. The cells were plated onto a COL1-coated container using a lower percentage of serum to minimize cell proliferation, as previously described [32]. Twenty-four hours after the scratch, the COL17-expressing cells exhibited significantly decreased migration compared to the non-producing cells (Figure 4C). To strengthen this result, we performed an invasion assay using basement membrane matrix Matrigel-coated chambers to mimic the *in vivo* extracellular environment [33]. Cells were allowed to invade depending on their invasive potential through membrane pores to the lower chamber for 24 hours. The invading cells were fixed, stained, and quantified. The COL17-expressing cells exhibited nearly 50% repressed invasive ability compared to mock cells (Figure 4D), suggesting a role of COL17 in suppressing cell migration and invasion.

Next, we examined the effect of the secreted form of COL17 on cells imitating the natural cell-matrix environment surrounding the mammary glands. Parental MDA-MB-231 cells were plated using conditioned media harvested from the stable cell culture dishes. The cells cultured in the COL17-enriched media demonstrated a significantly lower percentage of invasion compared to the cells cultured in mock media (Figure 4E), indicating that the COL17 ectodomain regulated cancer cell invasion. Our results established a role of COL17 in suppressing breast tumor migration and invasion *in vitro*.

### *COL17A1* depletion is associated with tumor progression and poor prognosis

**Figure 5:**
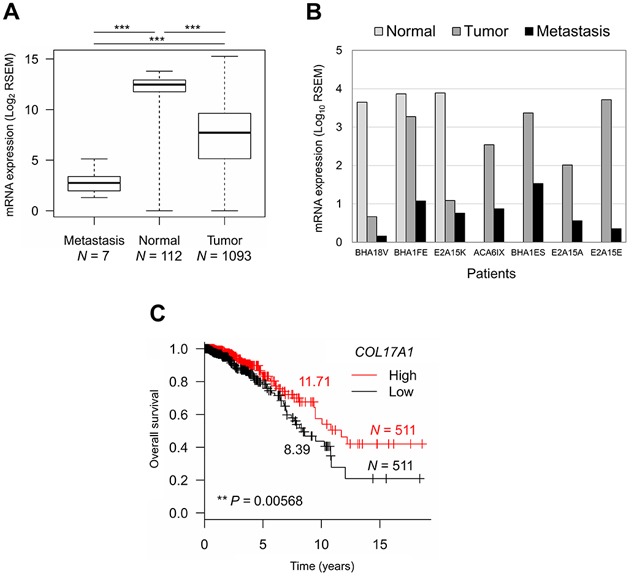
*COL17A1* depletion associates with tumor progression and poor prognosis **A.** and **B.** Differential mRNA expression of *COL17A1* in metastatic, normal, and tumor tissues from all patients **A.** and from the seven patients with metastasis **B.** *N*, number of tissues. Mann-Whitney *U*-test; *P < 0.05, **P < 0.01, ***P < 0.001. **C.** Kaplan-Meier survival curve of patients with high (red) and low (black) expression of *COL17A1* compared to median expression. Number, median survival time (years). *N*, number of patients. *P*-value was assessed by log-rank test.

We further analyzed the relationship between *COL17A1* expression and tumor progression in breast cancer patients using TCGA data. The *COL17A1* mRNA expression level was significantly decreased in metastatic tissues compared to primary tumors (Figure 5A) and even from the same patients (Figure 5B). Moreover, low *COL17A1* expression was significantly associated with a shorter median survival time among patients with breast invasive carcinoma (Figure 5C). In summary, we elucidated the role of COL17A1 as a breast tumor metastasis suppressor by inhibition of cancer cell migration and invasion, resulting in a better prognosis of patients.

## DISCUSSION

This study presents a p53-target screening based on human breast cell lines and breast cancer tissues. Ten genes including *COL17A1, CROT, FAM198B, GREB1, LAMA3, MICALCL, NPY1R, SLC27A2, SYTL2,* and *TSPAN1* are identified as p53 target gene candidates from this screening*.* GO analysis identified two enriched biological processes as significantly up-regulated by wild-type *p53*, epidermis development and ectoderm development, using a similar set of genes, i.e., *COL17A1, LAMA3,* and *EDA2R*. *LAMA3* encodes Laminin-5, an extracellular matrix protein, which binds to COL17 and functions in cell-matrix adhesion [34]. An alteration in the expression patterns of cell adhesion molecules, including Laminin-5, by knockdown and mutations of *p53* in MCF10A cells has been previously described, but COL17 has not been examined [23]. Here, we provide evidence that COL17 expression levels are elevated after the induction of endogenous wild-type *p53* following ADR-treatment in human breast cells or X-ray in mouse mammary glands. The results from a reporter assay and a ChIP assay indicate binding sites of p53 in intron 1, indicating *COL17A1* as a direct p53 target. In response to X-ray exposure, COL17 protein is expressed in the myoepithelial cells (basal cell) of the mammary gland, indicating its function in facilitating cell adhesion to the underlying basement membrane [15, 35]. Consistent with our result, gene expression profiling demonstrated that *COL17A1* expression is high in human breast myoepithelial cells but low in luminal epithelial cells [36].

The loss of function and mutation of p53 not only prevent breast cancer cells from undergoing oncogene-induced senescence and apoptosis but also result in the disruption of metastasis-involved molecules [7, 37, 38]. To metastasize, cells must invade through the basement membrane, enter the vasculature (intravasate), survive in the absence of adhesion, exit the vasculature (extravasate) and establish a new tumor in a foreign microenvironment [39]. The alteration and loss of cell adhesion structures are involved in an early step in epithelial cancer progression [40]. *COL17A1* expression is particularly low in metastatic breast cancer cells [17]. Conversely, MDA-MB-231 cells that genetically over-express COL17, exhibited significantly decreased invasive properties. The released COL17 ectodomain, also known as LAD-1, was detected in cell culture media, human skin extracts, and serum obtained from patients with an autoimmune blistering disorder [41]. The skin blistering of this disease is caused by autoantibodies against the COL17 ectodomain, which introduces its role in maintaining dermal-epidermal cohesion [42]. Recent studies have clarified its function in decreased keratinocyte migration by dampening mTOR signaling [18]. This study revealed the role of the 120-kDa COL17 ectodomain in the suppression of breast cancer cell invasion. Indeed, myoepithelial cells act as natural tumor suppressors by secreting various molecules that have inhibitory effects on tumor cell growth, invasion and angiogenesis [43]. Our results propose a new mechanism supporting myoepithelial cell function as a physical barrier to prevent the invasion of tumor cells from the duct to the stroma. An analysis of metastasis breast invasive carcinoma revealed that each patient demonstrates different expression levels of *COL17A1* in different tissue types depending on the tumor progression. Moreover, patients bearing high *COL17A1*-expressing tumors exhibit a longer survival time compared with those bearing low *COL17A1*-expressing tumors. Taken together, our results implicate a role of COL17 in suppressing breast cancer cell migration and invasion, whereby a high level of *COL17A1* expression leads to a better prognosis of patients with breast invasive carcinoma.

In this study, *COL17A1* was identified as a p53 transcriptional target gene. Wild-type p53 suppresses breast cancer invasion in pre-metastatic steps by up-regulating the expression of COL17, resulting in the promotion of the adhesion between the basement membranes and myoepithelial cells. The COL17-mediated adhesion prevents the primary tumor cells from escaping into the surrounding breast tissues. By contrast, p53 loss or mutation diminished COL17 function, resulting in less adhesive basement membranes, which cause it to eventually break down and allow tumor cells to escape into the bloodstream to form metastasis in the secondary organ, thereby contributing to a worse prognosis of patients. Although the molecules involved in the p53-COL17 signaling pathway are essential to be elucidated, our findings indicated the regulation of breast cancer metastasis by the p53-COL17 pathway and potential roles of COL17 as a prognostic biomarker.

## MATERIALS AND METHODS

### Cell lines and treatments

The non-tumorigenic breast epithelial cell line MCF10A *p53*^+/+^ and its isogenic *p53* knockout cell line (MCF10A *p53*^−/−^) were purchased from Sigma Aldrich (St. Louis, MO, USA). HBC4 cells were a gift from Dr. Takao Yamori (Japanese Foundation for Cancer Research, Tokyo, Japan). Human embryonic kidney cells transformed with the SV40 large T antigen (293FT) were provided in the ViraPower™ Lentiviral Expression System (Thermo Fisher Scientific). The other cell lines were purchased from the American Type Culture Collection (ATCC, Manassas, VA, USA). Cell cultures were maintained under their depositors’ recommendations. Cells were transfected with plasmids using FuGENE6 (Promega, Madison, WI, USA). siRNA oligonucleotides (see sequences in Supplementary Table 1), commercially synthesized by Sigma Genosis (St Louis, MO, USA), were reverse transfected with Lipofectamine RNAiMAX (Thermo Fisher Scientific) according to the manufacturer's protocol. To induce p53, cells were continuously incubated with ADR for 2 hours on the day following cell plating. Based on the cytotoxic effects of the drug, MCF10A, HBL-100, and HBC4 cells were treated with 0.5, 1, and 2 μg/mL of ADR, respectively.

### Microarray

Total RNA was isolated from MCF10A *p53*^+/+^ or MCF10A *p53*^−/−^ cells at 12, 24, and 48 hours after ADR treatment, as well as from non-treated cells as described [44]. The integrity and purity of the RNA templates were determined using a 2100 Bioanalyzer (Agilent Technologies). Agilent's One-Color Quick Amp labeling kit (Agilent Technologies) was used to generate fluorescent cRNA using cyanine 3-labeled targets according to the manufacturer's instructions. Gene expression analysis was performed using SurePrint G3 8×60K microarray (Agilent Technologies). The fold-change after ADR in the MCF10A *p53*^+/+^ cells was calculated using the following equation:

$$\text{Fold}=\frac{\text{Median expression of probe in [}p53^{+/+}\text{ ADR}+(12,24,48\text{ h)]}}{\text{Maximum expression of probe in [}p53^{+/+}\text{ ADR}-\text{and }p53^{-/-}\text{ all]}}$$

An *F*-test and two-tailed Student's *t*-test were performed to calculate the *P-*value. Genes demonstrating at least one probe satisfied the screening criteria, i.e., above 3-fold and *P* < 0.05, were included. The MCF10A microarray data is available from the NCBI GEO database (GSE98727).

### TCGA analysis

The mRNA expression of p53 target gene candidates, the *p53* mutation status, and clinical data were obtained from TCGA project by cBioPortal [45, 46]. For the differential expression analysis, 1093 tumor tissues were categorized according to *p53* mutation status and were subjected to a box plot analysis. The fold-change in wild-type *p53* tumors was calculated using the following equation:

$$\text{Fold}=\frac{\text{Median expression in [}p53\text{ wild-type tumors]}}{\text{Maximum expression in [}p53\text{ mutant tumors]}}$$

An *F*-test and two-tailed Student's *t*-test were performed to calculate the *P-*value as described [44]. Genes that satisfied the screening criteria, i.e., above 2-fold and *P* < 0.05, were included. A survival analysis was performed using the log-rank test stratified by the expression level of *COL17A1* in tumors (above or below the median expression level). Box plot and survival analyses were performed using the EZR software program [47].

### Gene Ontology (GO) enrichment analysis

The resultant 17 gene set was submitted for Gene Ontology analyses using the DAVID Functional Annotation Tool [ https://david.ncifcrf.gov/home.jsp]. The enrichment score for biological process was calculated using the chi-squared test.

### RNA extraction and quantitative PCR (qPCR)

Total RNA was isolated from cells and tissues using RNeasy Plus Mini Kits (Qiagen) according to the manufacturer's instructions. Complementary DNA molecules were synthesized using the SuperScript III reverse transcriptase (Invitrogen). qPCR was performed using SYBR Green Master Mix and a Light Cycler 480 (Roche). The primer sequences are shown in Supplementary Table 1.

### Western blot analysis

Cells were harvested by scraping and were lysed using chilled RIPA buffer (Thermo Fisher Scientific) containing 1 mM PMSF, 0.1 mM DTT, and 0.1% Protease Inhibitor Cocktail Set III (Calbiochem). The cell lysates were sonicated using a Bioruptor UCD-200 (Cosmobio, Tokyo, Japan) and centrifuged at 15,000 × *g* for 15 minutes at 4°C. Protein concentrations were measured using a BCA™ protein assay (Thermo Fisher Scientific). For media extract, cell culture media were changed to antibiotic-free media at 12 hours before harvest. Proteins were precipitated in chilled acetone, incubated for 1 hour at −80°C, and centrifuged at 15,000 × *g* for 15 minutes at 4°C. The cell lysates and precipitated protein from the media were added to SDS sample buffer (Bio-Rad), boiled for 5 minutes, and separated using SDS-PAGE. The proteins were transferred onto nitrocellulose membranes, which were subsequently blocked in 5% milk. The membranes were incubated with primary antibodies according to the manufacturers’ protocols. The membranes were incubated with horseradish peroxidase (HRP)-conjugated secondary antibodies for 1 hour at room temperature. The immunoblots were developed using Amersham™ ECL (GE Healthcare), and images were acquired using the luminescent image analyzer LAS-4000 mini (Fujifilm, GE Healthcare). The western blot signal representing protein level was obtained by image quantification using ImageJ software.

### Immunohistochemistry and immuno-cytochemistry

For immunocytochemistry, cells were plated onto glass coverslips, fixed with 4% paraformaldehyde, and permeabilized in 0.2% Triton-X in PBS. The samples were blocked with 3% BSA, stained with anti-Collagen XVII, incubated with HRP-conjugated secondary antibodies, and counterstained with DAPI. The mounted coverslips were visualized using a confocal microscope (Olympus FluoView FV1000). For immunohistochemistry, paraffin sections of mouse mammary tissues were stained using anti-Collagen XVII according to the manufacturer's protocol. For visualization, the sections were incubated with HRP-labeled polymer anti-rabbit (Dako) and DAB (Dako) was used as a chromogen. Then, the samples were counterstained with hematoxylin.

### Antibodies

Anti-Collagen XVII (ab184996, Abcam) was used for all human and mouse experiments. Anti-actin (AC15, Sigma-Aldrich), anti-p53 (OP43, Merck Millipore), and anti-p53-S15P (9284, Cell Signaling) were used in the western blot analyses.

### Animal experiments

The *p53* knockout C57BL/6J mice were provided by RIKEN BioResource Center (Ibaraki, Japan) [48]. The mice were maintained under specific pathogen-free conditions and were handled according to the Guidelines for Animal Experiments of the University of Tokyo. The mouse genotypes were confirmed by PCR analysis. Primer sequences are shown in Supplementary Table 1. The *p53* wild-type and knockout female mice at 10 weeks of age were exposed to 10 Gy of X-rays using an X-ray irradiation system (MBR-1520R-3, Hitachi). At 24 hours after irradiation, the mice were sacrificed for mammary tissue extraction.

### Gene reporter assay

A DNA fragment that includes the potential p53 binding sites of *COL17A1* was amplified and subcloned into the pGL4.24 [*luc2P*/minP] vector (Promega). To create a mutant vector, point mutations were introduced at the 4^th^ and 14^th^ nucleotides (C to T mutations) and the 7^th^ and 17^th^ nucleotides (G to T mutations) within the consensus p53 binding site using site-directed mutagenesis. Reporter assays were performed using the Dual-Glo Luciferase Assay System (Promega) according to the manufacturer's protocol. The H1299 cells were co-transfected with the analyzed constructs and the control vector (pcDNA3.1), wild-type p53 (p53), or mutant p53 (p53R175H). The primer sequences for amplification and site-directed mutagenesis are shown in Supplementary Table 1.

### Chromatin immunoprecipitation (ChIP) assay

ChIP assays were performed using EZ-Magna ChIP G Chromatin Immunoprecipitation Kits (Merck Millipore, Darmstadt, Germany) according to the manufacturer's protocol. Briefly, HBC4 cells with or without ADR treatment were cross-linked with 1% formaldehyde for 10 minutes, washed with PBS, and lysed in nuclear lysis buffer. The lysate was then sonicated using Bioruptor UCD-200 (Cosmo Bio, Tokyo, Japan) to shear the DNA into fragments of approximately 200-1000 bp. The supernatant from 1 × 10^6^ cells was used for each immunoprecipitation with anti-p53 antibody (OP140, Merck Millipore) or mouse IgG (SC-2025, Santa Cruz). Before immunoprecipitation, 1% of the supernatant was removed as “input”. Column-purified DNA was quantified by qPCR using primers for p53 binding site in *WAF1* and *COL17A1* BS primers (Supplementary Table 1).

### Stable cell line generation

The full-length *COL17A1* genomic DNA fragment containing Kozak sequence was cloned into the entry vector (pENTR™3C). The primer sequences are shown in Supplementary Table 1. The *COL17A1* fragment was integrated into the destination vector (pLenti6.3/TO/V5-DEST) using Gateway LR Clonase II enzyme mix and then transformed into Stbl3 cells to generate the expression clone. Lentiviruses containing *COL17A1* expression construct, empty vector, or *TetR* vector (pLenti3.3/TR) were produced in 293FT cells using ViraPower™ Packaging Mix according to the manufacturer's protocol. The virus-containing media were harvested and used to determine the viral titer by qPCR. The *TetR* construct was co-transduced at 10 MOI into MDA-MB-231 cells with either the *COL17A1* construct for COL17A1-expressing cells or empty vector for mock cells. The transduced cells were selected and continuously cultured in media containing Blasticidin and Geneticin^®^ for 3 weeks. Stable cells were maintained using Leibovitz's L-15 medium in a humidified 37°C incubator without CO_2_.

### Scratch assay

Stable cells were treated with 1 μg/mL doxycycline (Dox) for 48 hours before cell plating. The cells (1.2 × 10^5^) were seeded onto the COL1-coated 24-well plates using 1% FBS-containing culture media with or without Dox. Twenty-four hours after plating, the confluent cells were scraped using a CELL Scratcher (Iwaki, Japan), washed and replaced with media. The culture plates were marked as reference points close to the scratch. Images of the scratched area at the reference points were recorded immediately after the scratch and 24 hours later using a phase-contrast microscope with 10× magnification. The distances of the scratched area were determined and measured using ImageJ software [49]. The average migration distance of each well was calculated and subjected to a box plot analysis using R programming.

### Invasion assay

Stable cells were treated with 1 μg/mL Dox for 48 hours before cell plating. The cells (7.5 × 10^4^) were seeded into the 24-well BioCoat Matrigel invasion chambers (Corning) or control inserts according to the manufacturer's protocol. The upper chamber filled with serum-free DMEM was placed in a well containing 10% FBS DMEM with the same condition of Dox, i.e., presence or absence. For the cell assay, stable cell lines were used with fresh media in both the upper chamber and the lower well. For the media assay, parental MDA-MB-231 cells were seeded into the chamber using conditioned media harvested from the stable cell culture dishes; and the chambers were placed in fresh media. After 24 hours of incubation, the cells invading to the bottom surface of the chamber membrane were fixed with 4% paraformaldehyde for 30 minutes and stained with 0.1% crystal violet for 2 h. The invading cells were counted using CELLCOUNTER software [50] and calculated as the percent invasion through the Matrigel relative to the control. The invasion index is displayed as the ratio of % invasion of Dox+ over those of Dox− condition.

## SUPPLEMENTARY FIGURES AND TABLE



## Abbreviations

ADR: Adriamycin^®^ (doxorubicin)
COL17: Collagen XVII
Dox: doxycycline
GO: Gene Ontology
HD: hemidesmosome
TCGA: The Cancer Genome Atlas
p53: tumor protein 53
