# Supplementary material for: Identification of a novel p53 target, COL17A1, that inhibits breast cancer cell migration and invasion
Source: Oncotarget. 2017 Jun 9;8(34):55790–803. doi: 10.18632/oncotarget.18433 (PMC5593524; doi:10.18632/oncotarget.18433)
Supplement: Supplementary file 1 [file oncotarget-08-55790-s001.pdf]

Identification of a novel p53 target, COL17A1, that inhibits breast cancer cell migration and invasion

Supplementary Material

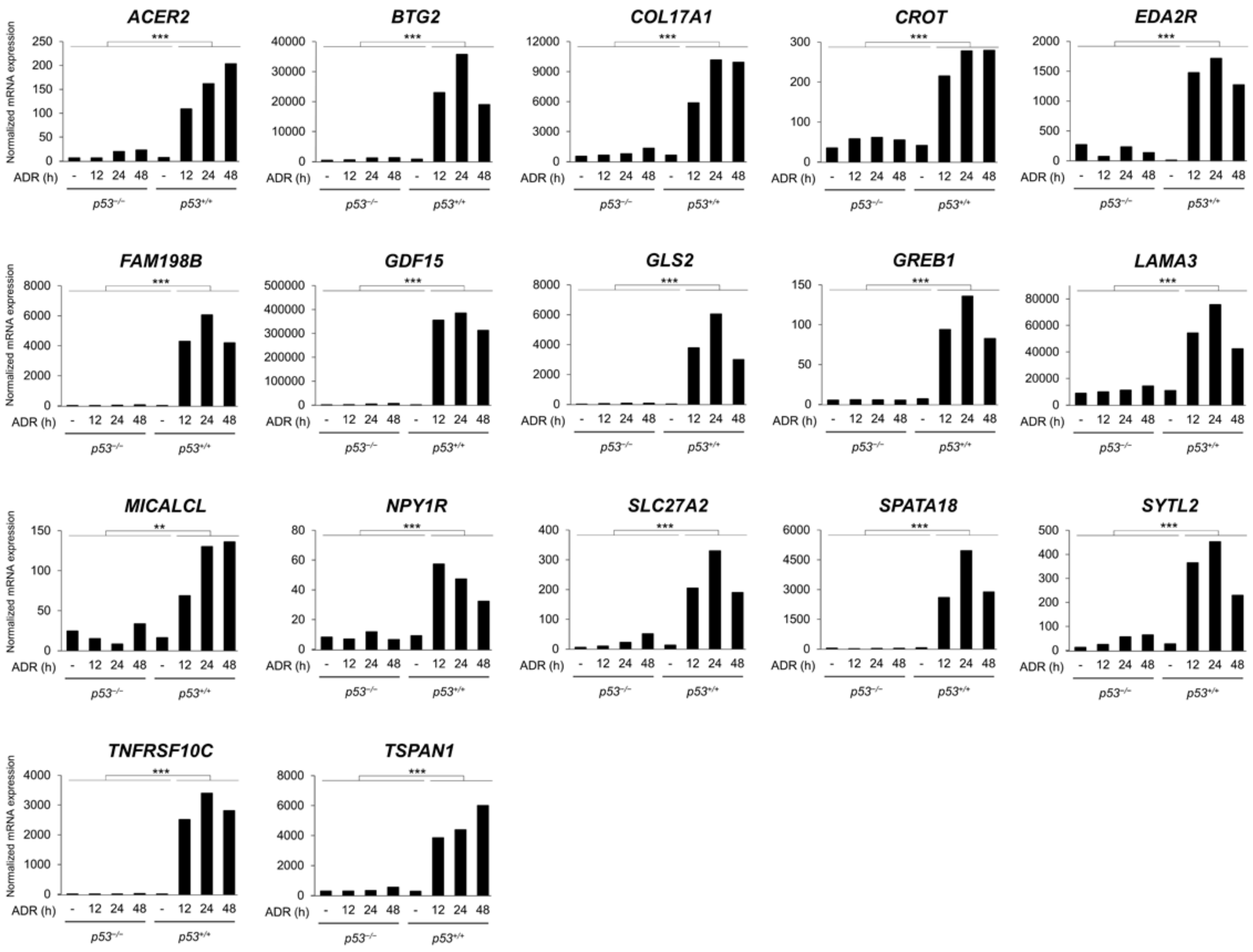

Supplementary Figure 1: MCF10A microarray results of the 17 p53 target gene candidates. Normalized expression of 17 genes' criteria-satisfied probes obtained from the cDNA microarray of MCF10A cells bearing *p53* wild-type ($p53^{+/+}$) or knockout ($p53^{-/-}$), with or without ADR; times (hours) indicate the period after ADR treatment. Two-tailed Student's *t*-test; *$P < 0.05$, **$P < 0.01$, ***$P < 0.001$.

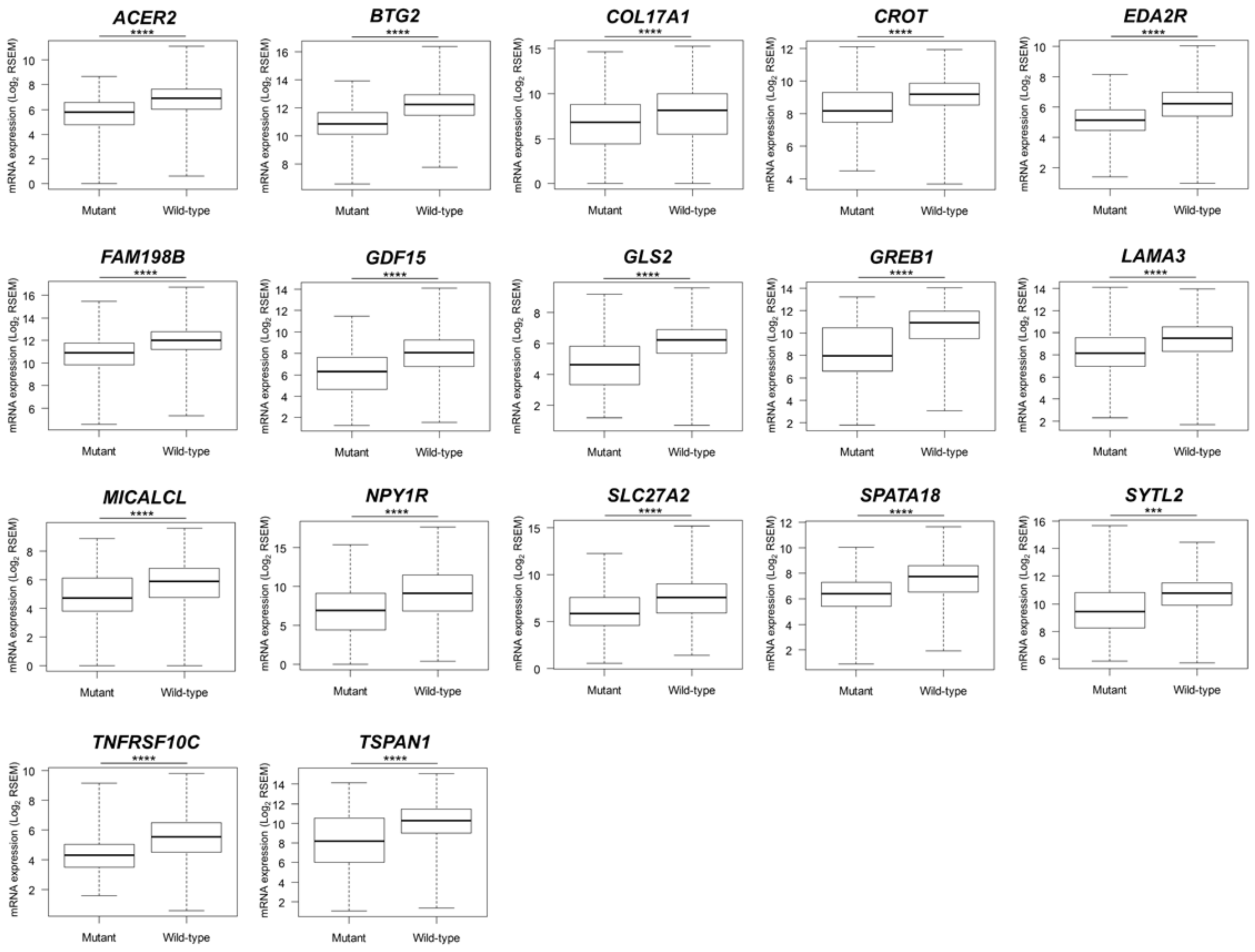

Supplementary Figure 2: TCGA results of the 17 p53 target gene candidates. Differential mRNA expression of 17 genes in *p53* wild-type and mutant tumors. Number of tissues; 298 *p53* mutant tumors, and 795 *p53* wild-type tumors. Two-tailed Student's *t*-test; $*P < 0.05$, $**P < 0.01$, $***P < 0.001$, $****P < 0.00024$ (0.05/209).

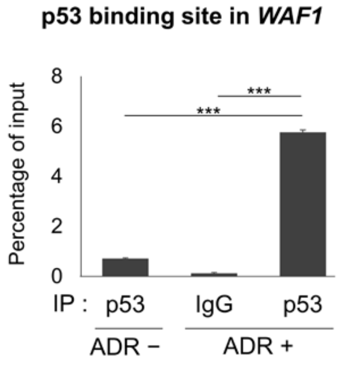

Supplementary Figure 3: ChIP assay of p53 binding site in WAF1. ChIP assay of non-treated or ADR-treated HBC4 cells of which DNA–protein complexes were then immunoprecipitated with the indicated antibodies followed by qPCR. Anti-mouse IgG was used as a negative control. The graph shows qPCR result indicating the amount of genomic fragments containing p53-binding sequence in *WAF1*, a p53 target gene. Two-tailed Student's *t*-test; $*P < 0.05$, $**P < 0.01$, $***P < 0.001$.

Supplementary Table 1: Sequences of DNA and RNA oligonucleotides

**siRNA oligonucleotides**	**Sense**	**Antisense**
siEGFP	GCAGCACGACUUCUUCAAGT	CUUGAAGAAGUCGUGCUGC
sip53	GACUCCAGUGGUAAUCUACTT	GUAGAUUACCACUGGAGUCTT
Primers	Forward	Reverse
Quantitative PCR		
Human COL17A1	CTGACTTTGCTGGAGATCTGG	TAGGCCATCCCTTGCAGTAG
Human B2M	TTCTGGCCTGGAGGCTATC	TCAGGAAATTTGACTTTCCATTC
Human ACTB	CCCTGGAGAAGAGCTACGAG	TGAAGGTAGTTTCGTGGATGC
Mouse Col17a1	GAAAGGAGACAAAGGTGACCA	CGGCTTGATGGCAATACTTC
Mouse Gapdh	AATGTGTCCGTCGTGGATCTGA	GATGCCTGCTTCACCACCTTCT
Mouse genotyping		
Mouse p53	GTTATGCATCCATACAGTACA	CCGCAGGATTTACAGACACC
Gene reporter assay		
BS: p53 binding site	TGGAAGACGAACACACTGGT	AGGTACGTGTTGGGAGACTG
MT1: mutated BS1	GTCTCAGGTATTTACCTGGGCAG GAAACGTTCC	TACCTATCATAACAGGATTCACCA GTGTGTTCGTCTTC
MT2: mutated BS2	AGGTTTTTGCCCCACGC	GGAATAAGATCTTCCCATGGGAC
ChIP assay		
p53 binding site in WAF1	CTGGACTGGGCACTCTTGTC	CTCCTACCATCCCCTTCCTC
COL17A1 BS	TGGAAGACGAACACACTGGT	AAATGGAGAGTGATGGCGTG
Stable cell line generation		
COL17A1 genomic DNA	GCCGCCATGGATGTAACCAAGAA AAACAAAC	TCACGGCTTGACAGCAATACTTC
